# Bronchoscopic needle-based confocal laser endomicroscopy (nCLE) as a real-time detection tool for peripheral lung cancer

**DOI:** 10.1136/thoraxjnl-2021-216885

**Published:** 2021-06-25

**Authors:** Tess Kramer, Lizzy Wijmans, Martijn de Bruin, Ton van Leeuwen, Teodora Radonic, Peter Bonta, Jouke T Annema

**Affiliations:** 1 Respiratory Medicine, Amsterdam University Medical Centers, Amsterdam, Noord-Holland, The Netherlands; 2 Department of Biomedical Engineering and Physics, Amsterdam University Medical Centers, Amsterdam, Noord-Holland, The Netherlands; 3 Department of Pathology, Amsterdam University Medical Centers, Amsterdam, Noord-Holland, The Netherlands

**Keywords:** bronchoscopy, imaging/CT MRI etc, lung cancer, non-small cell lung cancer, histology/cytology

## Abstract

**Introduction:**

Diagnosing peripheral lung cancer with the bronchoscope is challenging with near miss of the target lesion as major obstacle. Needle-based confocal laser endomicroscopy (nCLE) enables real-time microscopic tumour visualisation at the needle tip (smart needle).

**Aim:**

To investigate feasibility and safety of bronchoscopic nCLE imaging of suspected peripheral lung cancer and to assess whether nCLE imaging allows real-time discrimination between malignancy and airway/lung parenchyma.

**Methods:**

Patients with suspected peripheral lung cancer based on (positron emission tomography-)CT scan underwent radial endobronchial ultrasound (rEBUS) and fluoroscopy-guided flexible bronchoscopy. After rEBUS lesion detection, an 18G needle loaded with the CLE probe was inserted in the selected airway under fluoroscopic guidance. The nCLE videos were obtained at the needle tip, followed by aspirates and biopsies. The nCLE videos were reviewed and compared with the cytopathology of the corresponding puncture and final diagnosis. Five blinded raters validated nCLE videos of lung tumours and airway/lung parenchyma twice.

**Results:**

The nCLE imaging was performed in 26 patients. No adverse events occurred. In 24 patients (92%) good to high quality videos were obtained (final diagnosis; lung cancer n=23 and organising pneumonia n=1). The nCLE imaging detected malignancy in 22 out of 23 patients with lung cancer. Blinded raters differentiated nCLE videos of malignancy from airway/lung parenchyma (280 ratings) with a 95% accuracy. The inter-observer agreement was substantial (κ=0.78, 95% CI 0.70 to 0.86) and intra-observer reliability excellent (mean±SD κ=0.81±0.05).

**Conclusion:**

Bronchoscopic nCLE imaging of peripheral lung lesions is feasible, safe and allows real-time lung cancer detection. Blinded raters accurately distinguished nCLE videos of lung cancer from airway/lung parenchyma, showing the potential of nCLE imaging as real-time guidance tool.

**Key messages**

**What is the key question?**

- Does bronchoscopic needle-based confocal laser endomicroscopy (nCLE) imaging allow real-time peripheral lung cancer detection?

**What is the bottom line?**

- In this prospective proof of principle study, we demonstrated for the first time that bronchoscopic nCLE imaging of peripheral lung lesions suspected of lung cancer is feasible, safe and enables real-time malignancy detection at the needle tip. Blinded raters were able to consistently distinguish nCLE videos of lung cancer from airway/lung parenchyma with high accuracy (95%) after a short training. The results of this study demonstrate the potential of nCLE imaging as a real-time bronchoscopic guidance tool to reduce the current near miss rate of sampling peripheral lung lesions.

**Why read on?**

- Our study provides novel information on how to improve peripheral lung cancer diagnostics by reducing the current substantial near-miss rate of the target lesion.

## Introduction

Lung cancer is the leading cause of cancer related deaths worldwide with annual 2 million incident cases and 1.7 million deaths.^1^ The growing use of chest CT and the expected implementation of low-dose CT screening for lung cancer, will lead to an increasing detection rate of lesions suspected of lung cancer that require further evaluation.^2^ Since tissue acquisition for pathological analysis is prerequisite for diagnosis and optimal treatment, a drastic increase in the number of bronchoscopies is expected.

Over 70% of the lesions suspected of lung cancer develop in the periphery of the lung.^3^ Although bronchoscopic guidance tools such as radial endobronchial ultrasound (rEBUS), fluoroscopy, electromagnetic navigation, cone beam CT and robotic bronchoscopy demonstrate an improved approximation of the peripheral lung lesion^4–11^ near miss of the target lesion remains a major obstacle.^12^ Therefore, bronchoscopic lung biopsies without tissue proof of malignancy are often considered to be non-diagnostic rather than non-malignant and additional (more invasive) procedures are indicated. Consequently, the need for a complementary bronchoscopic guidance tool that provides real-time feedback on the correct needle positioning is urgent.

Confocal laser endomicroscopy (CLE) is a high resolution laser-based imaging technique that is performed with intravenous administration of the contrast agent fluorescein to visualise individual cells and structures. Recently, the CLE probe has become small enough to fit through a biopsy needle (needle-based CLE (nCLE)), enabling real-time in-vivo microscopic analysis at the needle tip (smart needle principle).^13–16^ For a reliable CLE smart needle, clear distinction between malignant tissue and adjacent airway or lung parenchyma is essential to reduce the near-miss rate of peripheral lung cancer. The nCLE criteria for malignancy (enlarged pleomorphic cells, dark clumps and directional streaming) in central lung tumours and metastatic lymph nodes have been identified^16^ but are lacking for airway and lung parenchyma.

Currently, feasibility, safety and interpretability of nCLE imaging of peripheral lung lesions is not known. In this proof of principle study, we hypothesise that bronchoscopic nCLE imaging of peripheral lung lesions is feasible, safe and allows real-time malignancy detection. Additionally, we aim to evaluate nCLE imaging as a real-time guidance tool by identifying nCLE criteria for airway/lung parenchyma and assessing whether blinded raters can differentiate malignancy from airway/lung parenchyma on nCLE imaging.

## Methods

### Study design

This is a prospective proof of principle study, conducted between May 2019 and July 2020 in the Amsterdam University Medical Centers, the Netherlands. In the initial design of the study we aimed to perform nCLE imaging both in conjunction with flexible bronchoscopy for peripheral lung lesion analysis and linear EBUS for mediastinal lymph nodes analysis. In the absence of EBUS needles that could accommodate the CLE probe, we only performed nCLE imaging in conjunction with flexible bronchoscopy for peripheral lung lesion analysis. Patients with a peripheral lung lesion suspected of lung cancer based on (positron emission tomography-)CT scan were considered eligible for study inclusion. Exclusion criteria were: <18 years of age, known allergy to fluorescein, pregnancy or lactating. Since beta-blockers are known to increase the anaphylaxis risk of intravenous fluorescein use,^17^ patients with beta-blocker use <12 hours prior to the procedure were excluded from study participation. All included patients provided written informed consent. Patients underwent radial EBUS and fluoroscopy-guided flexible bronchoscopy as part of routine clinical care. For study purposes, nCLE imaging of the lesion was performed at the needle tip followed by transbronchial needle aspiration (TBNA) and biopsies.

Feasibility of bronchoscopic nCLE-imaging was defined as acquiring adequate real-time CLE video footage of the target lesion in >80% of cases. Safety was defined as the absence of adverse events related to the nCLE measurements and fluorescein use. To evaluate the nCLE malignancy detection rate, in-vivo nCLE videos were compared with the final diagnosis using published pre-defined nCLE malignancy criteria.^16^ The final diagnosis was based on the (cyto-)pathology results of TBNA and forceps biopsies or in case of negative bronchoscopic tumour sampling, surgical–pathological staging or clinical–radiological follow-up at 6 months.

**Figure 1:**
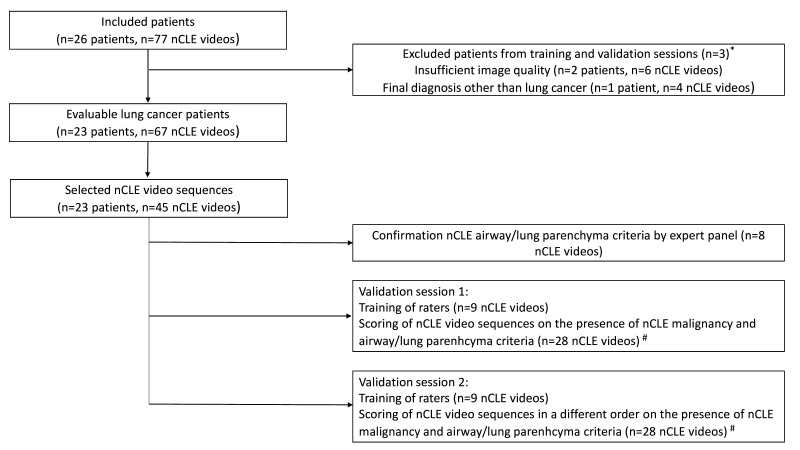
Study flow diagram. *In total, three patients (10 nCLE videos) were excluded from the video selection of the training and validation sessions due to poor nCLE image quality and a non-malignant final diagnosis (organising pneumonia). ^#^Of every nCLE-imaged lung tumour at least one video was included in the validation set to make the selection most representative. nCLE, needle-based confocal laser endomicroscopy.

In order to assess nCLE malignancy and airway/lung parenchyma discrimination accuracy, this study was divided into three steps (figure 1). Step 1 aimed to identify nCLE airway and lung parenchyma criteria. In step 2, blinded raters were trained in the predefined nCLE malignancy and novel airway/lung parenchyma criteria, directly followed by reviewing of nCLE videos on the presence of these criteria. A second identical training and validation session took place after a 2-week wash-out period with the same nCLE videos presented in a different order (step 3). In order to make the validation sessions feasible in time, nCLE videos used for steps 2 and 3 were preselected sequences with an average duration of 45 s (figure 1). Of every evaluable nCLE-imaged lung tumour at least one video was included in the validation set.

### Study procedure

**Figure 2:**
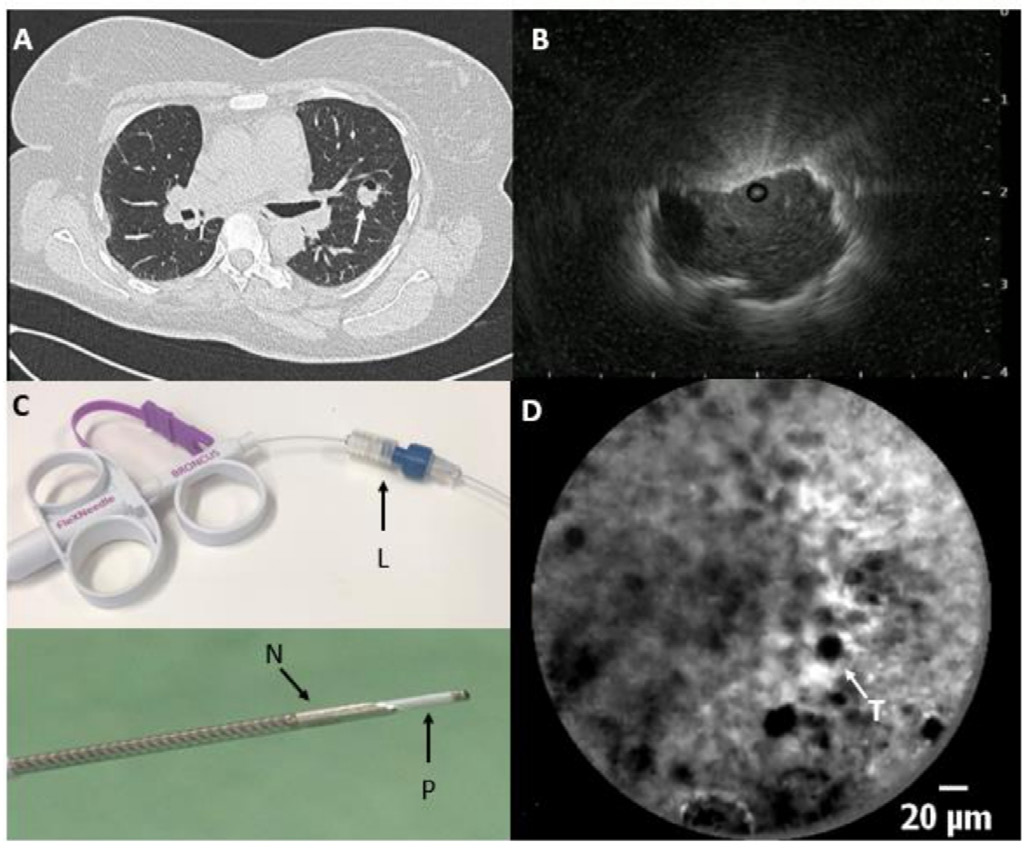
(A) Chest CT scan with a lung tumour in the left upper lobe (arrow) and (B) radial endobronchial ultrasound image with an eccentric tumour visualisation. (C) Preloading of the needle:after adjusting the luer lock (L) on the 18G Broncus needle, the confocal miniprobe is advanced through the luer lock, positioning the tip of the probe (P) 4 mm past the needle tip (N). (D) In-vivo needle-based confocal laser endomicroscopy image at the tip of the needle showing real-time pleomorphic enlarged tumour cells (T) representing a sarcoma metastasis.

Patients with a suspected malignant peripheral lung tumour and referred for bronchoscopic analysis, were screened and enrolled in the study provided that the bronchoscopist had sufficient confidence on reaching the lesion to attempt a needle puncture pass. Two bronchoscopists (JTA and PB) performed the procedures with the patient under deep sedation. The nCLE videos were obtained using the Cellvizio system and the AQ-Flex 19 miniprobe with an external diameter of 0.85 mm and a resolution of 3.5 μm (Mauna Kea Technologies, Paris, France). The diameter of the probe is compatible with the diameter of the 18G-Flex needle (Broncus Medical, Seattle, USA). Prior to bronchoscopy, an 18G needle was preloaded with the CLE miniprobe using a locking device (figure 2). The procedure started with a bronchoscopic inspection of the airways to exclude endobronchial abnormalities. Based on the pre-procedural CT scan was decided which airways had the best chance on reaching the tumour lesion. Subsequently the rEBUS miniprobe was advanced in the selected airway and if the tumour was visualised on rEBUS, the rEBUS probe positioning on the fluoroscopy was checked. The rEBUS probe was then removed from the working channel of the bronchoscope and the needle, containing the preloaded CLE probe, was advanced in the selected airway. No guide sheath was used. Lesion puncture was performed at the previously decided sampling location using fluoroscopic guidance.

Right before nCLE imaging, fluorescein (2.5 mL of 10% fluorescein dinatrium solution) was administered intravenously. After lesion puncture and fluorescein administration, the CLE miniprobe was advanced in a forward direction, securely positioning the miniprobe past the needle tip with the locking device. Based on real-time nCLE imaging, the optimal TBNA/biopsy location was identified and localised on the fluoroscopy. After nCLE imaging, the CLE miniprobe was retracted, followed by aspiration of tumour cells at the same spot using suction. Every nCLE video was followed by a separate lesion puncture, enabling direct correlation between the nCLE videos and the cytology results of the corresponding needle aspirate. The cytology specimen was prepared on glass-slides with H&E staining and cell blocks were obtained. Rapid on site evaluation was available and was part of the clinical decision-making how many TBNA-nCLE passes were performed. Following TBNA, forceps biopsies were taken under fluoroscopic control.

### nCLE analysis

**Figure 3:**
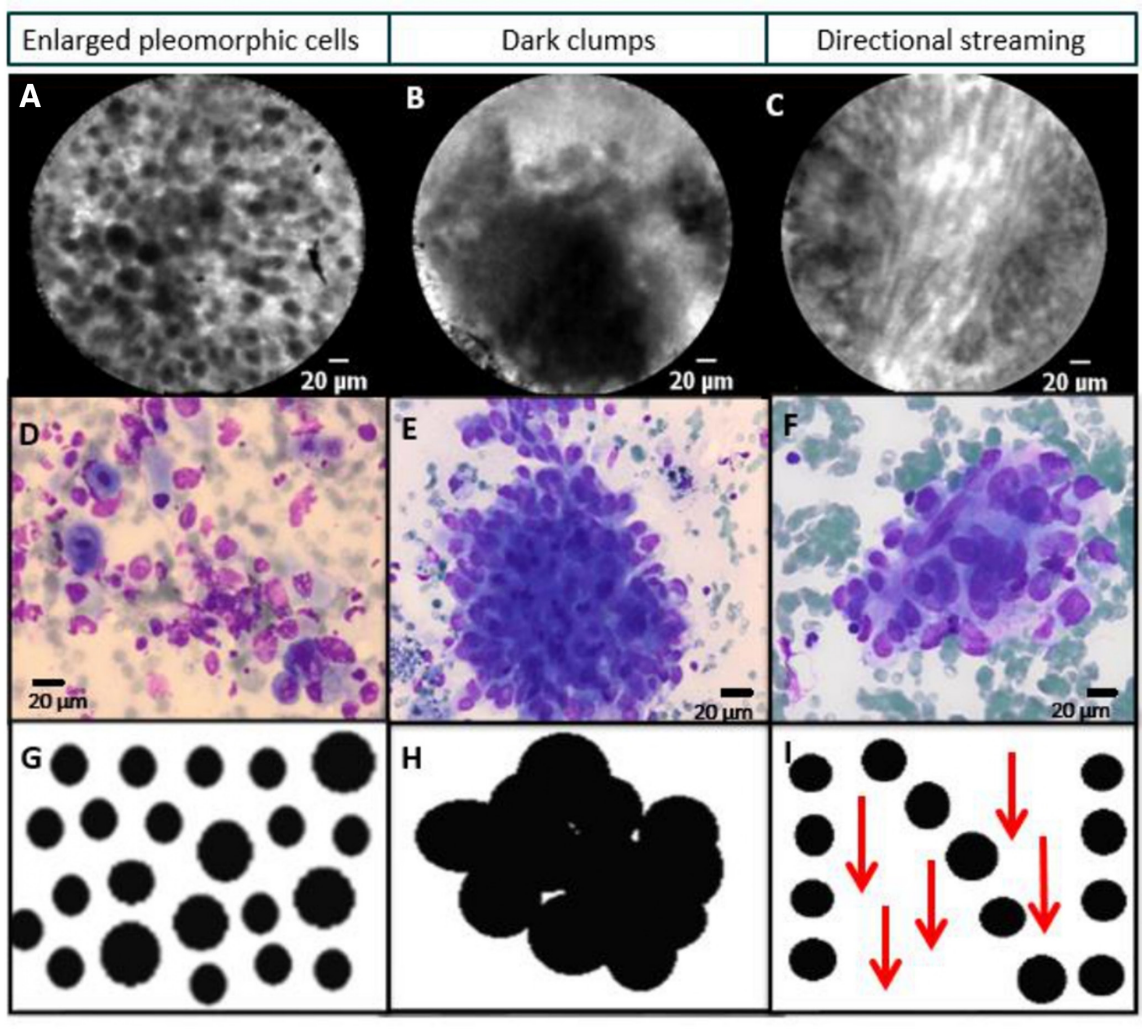
(A–C) Real-time needle-based confocal laser endomicroscopy (nCLE) imaging of different lung tumours demonstrating the two ‘static’ nCLE malignancy criteria (enlarged pleomorphic cells and dark clumps) and the ’dynamic’ phenomenon of directional streaming (online example). (D–F) Corresponding cytology of the fine needle aspirate representing squamous cell carcinoma, adenocarcinoma and sarcoma metastasis. (G–I) Schematic display of malignant nCLE features.

All nCLE videos were reviewed on the presence of malignant criteria and compared with the final diagnosis and corresponding cytopathology. At least one of the three nCLE lung cancer criteria (enlarged pleomorphic cells, dark clumps or directional streaming (figure 3)) had to be visualised to confirm the presence of malignancy. Based on nCLE videos without malignant features and the absence of malignant or atypical cells in the cytopathology of the corresponding puncture, novel nCLE airway/lung parenchyma criteria were proposed and confirmed by an expert panel (two pulmonologists, CLE researcher and pathologist). The nCLE videos used for confirmation of the identified airway/lung parenchyma criteria were excluded from the training and validation sessions in order to prevent selection bias.

**Figure 4:**
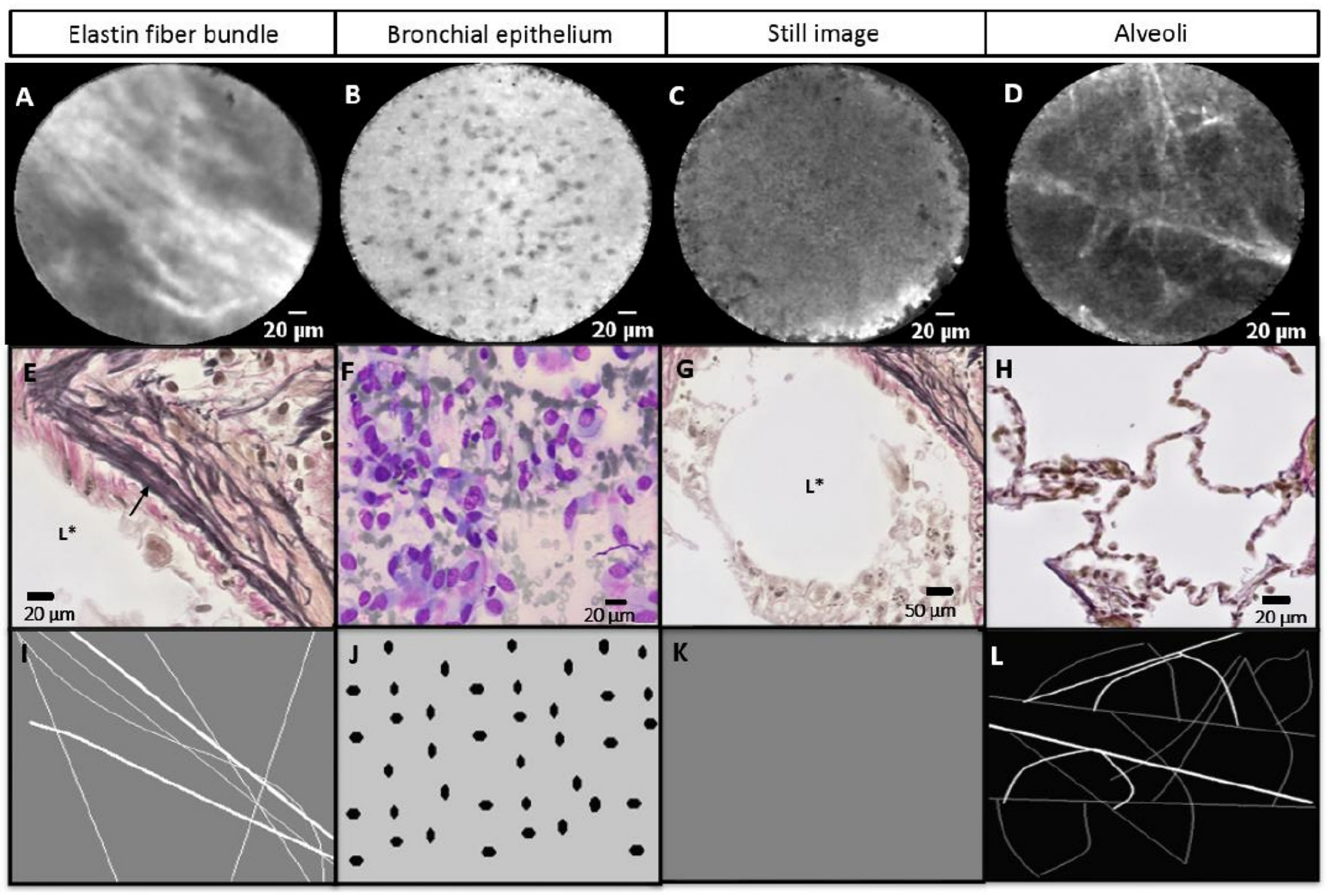
(A–D) Real-time needle-based confocal laser endomicroscopy (nCLE) imaging at the needle tip showing three nCLE airway criteria (elastin fibres, bronchial epithelium and still image) and the alveoli of the lung parenchyma. (E–H) Histology (E, G and H) and cytology (F) of the different structures in the airway and lung parenchyma. (I–L) Schematic display of the airway (I–K) and lung parenchyma (L) nCLE features. (A) Autofluorescent elastin fibre bundles (indicated in E by arrow) along the lumen of the airway (indicated in E by L*). (B) Small homogeneous and equally distributed cells representing the bronchial epithelium (F). (C) Still nCLE image as the result of the CLE-probe being advanced in the lumen of a larger airway (indicated in G by L*) without touching the airway wall. (D) Autofluorescent alveoli with a hexagonal architecture (H). Scale bar: (A–F and H) 20 µm and (G) 50 µm.

#### Training session

During a training of approximately 15 min, five raters (two pulmonologists, two pulmonology residents and one pulmonology researcher) were trained in published nCLE lung cancer^16^ and novel proposed airway/lung parenchyma criteria (figures 3 and 4) using PowerPoint slides, hand-drawn schematics and a preselected set of training videos (figure 1). None of the raters were involved in the study design or data acquisition. All raters were inexperienced with CLE imaging, except for one rater who is involved in CLE imaging in interstitial lung disease. The raters received prints of the hand-drawn schematics which could be used during the validation sessions (figures 3 and 4).

#### Validation session

Directly after training, the first validation session took place. Raters, blinded for the cytopathology results, independently scored the videos on the individual presence of the three malignancy criteria and four airway/lung parenchyma criteria (yes/no). The rater’s final nCLE diagnosis was established by assigning the dominant pattern (tumour or airway/lung parenchyma) and level of confidence (good/moderate/poor). Videos used for the training session were excluded. The second validation session was performed after a 2-week wash-out period with the nCLE videos in a different order to assess the intra-observer reliability (IOR).

### Statistical analysis

The sensitivity, specificity, positive predictive value, negative predictive value, accuracy and the IOR were calculated using the standard definitions and software from the SPSS statistical package V.25.0 (IBM Corporation). The accuracy of the performance of the raters was calculated using the scores of the five raters in both validation sessions (total of 280 ratings).

The inter-observer agreement (IOA) was calculated with MATLAB R2020b (MathWorks, Natick, Massachusetts, USA) using multi-rater Fleiss’ κ.^18^ The IOR was calculated using Cohen’s κ by comparing the results between the first and the second session. The results of the IOR and IOA were interpreted according to the Landis-Koch interpretation system: poor <0.2, fair 0.21–0.4, moderate 0.41–0.6, substantial 0.61–0.8 and excellent 0.81–1.^19^

## Results

### Patient characteristics

Bronchoscopic nCLE imaging was performed in 26 patients with suspected peripheral lung cancer. In 24 out of 26 patients (n=71 out of 77 nCLE videos, 92%) adequate nCLE videos were obtained. Two patients (n=6 nCLE videos, 8%) were excluded from analysis because of inadequate nCLE imaging due to staining of the probe or improper contact between the probe and the tissue. No adverse events related to the nCLE imaging or fluorescein administration occurred.

Patient characteristics are displayed in table 1 and final diagnoses were adenocarcinoma (n=12), squamous cell carcinoma (n=6), non-small cell lung cancer not further specified (n=1), metastasis of urothelial (n=1), sarcoma (n=1), endometrium (n=1) and oesophagus carcinoma (n=1), organising pneumonia (n=1). Bronchoscopic lesion sampling, including forceps biopsies, detected malignancy in 20 out of 24 patients (n=23 with lung cancer; n=1 organising pneumonia). In one patient, no cytological punctures of the tumour were taken because of desaturations during the procedure and the nCLE imaging was compared with the histology results of the biopsy. Three patients with a false-negative bronchoscopic tumour sampling underwent additional procedures (transthoracic CT-guided biopsy, lobectomy or paravertebral biopsy) and were diagnosed with adenocarcinoma. On average three punctures with corresponding nCLE imaging were performed per patient, resulting in a total of 71 nCLE videos.

### nCLE criteria

#### nCLE malignancy detection

nCLE imaging detected a malignant nCLE pattern (presence of at least one nCLE malignancy criterium) in 22 out of 23 patients with lung cancer, including two patients with a false-negative tumour sampling. In one patient, nCLE imaging did not detect malignancy and an alveolar pattern was visualised.

**Table 1:** Patient characteristics and final diagnoses

Variable	Value
Evaluable patients	24
Age years (mean, SD)	64.1, ±10.3
Sex	
Male	12
Female	12
Final diagnosis	
Non-small cell lung cancer	19
Adenocarcinoma	12
Squamous cell carcinoma	6
Not further specified	1
Metastasis urothelial carcinoma	1
Metastasis sarcoma	1
Metastasis endometrium carcinoma	1
Metastasisoesophagus carcinoma	1
Organising pneumonia	1
Lesion localisation	
Right upper lobe	8
Right middle lobe	1
Right lower lobe	4
Left upper lobe	8
Left lower lobe	3
Lesion size (mean largest diameter in mm, range)	41, 11–89
Bronchus sign	
Present	23
Absent	1

The three predefined malignant nCLE criteria were observed in all different subtypes of lung cancer (figure 3). The malignant feature ‘enlarged pleomorphic cells’ was consistently present in all nCLE-imaged lung tumours (n=22 patients, n=50 nCLE videos), while dark clumps (n=18 patients, n=30 nCLE videos) and directional streaming (n=16 patients, n=25 nCLE videos) were variably present. In three patients with adenocarcinoma, necrosis caused a loss of contact signal which could be visualised as a crosshatch pattern; this phenomenon is not specific for adenocarcinoma. A distinct nCLE pattern was observed in four patients showing dark clumps of tumour cells lining the alveolar wall, matching histology revealed lepidic growing adenocarcinoma. In the online supplemental nCLE-video all three nCLE malignancy criteria are demonstrated.

10.1136/thoraxjnl-2021-216885.supp1 Supplementary video

#### nCLE airway/lung parenchyma detection

In 14 out of 67 (21%) TBNA samples of patients with lung cancer, no malignancy was detected in both the nCLE imaging and the cytopathology of the corresponding puncture. Based on these videos, four nCLE criteria for guiding airways and lung parenchyma were proposed: (1) ‘elastin fibre bundle’ and (2) ‘still image without cellular structures’ representing conducting airway, (3) ‘small, homogeneous cells’ representing the bronchial epithelium and (4) ‘autofluorescent alveoli networks’ representing the lung parenchyma (figure 4).

After identification of novel airway/lung parenchyma criteria, nCLE videos with a malignant diagnosis were reviewed and in 12 nCLE videos a combination of malignancy and airway/lung parenchyma criteria was present, resulting in a total of 26 nCLE videos with airway and lung parenchyma visualisation. Of the airway and lung parenchyma criteria, the criterium ‘elastin fibre bundle’ was most present (n=11 patients, n=13 nCLE videos) followed by ‘bronchial epithelium’ (n=11 patients, n=12 nCLE videos), ‘still image’ (n=9 patients, n=9 nCLE videos) and ‘alveoli’ (n=3 patients, n=4 nCLE videos).

### nCLE malignancy and airway/lung parenchyma discrimination

The blinded raters scored the nCLE videos on the presence of malignancy and airway/lung parenchyma criteria with an overall sensitivity and specificity of 0.96 (95% CI 0.89 to 1.0) and 0.94 (95% CI 0.85 to 1.0), respectively (table 2). Of the nCLE videos that were rated with ‘good’ (n=198; 70.7%) or ‘moderate’ (n=62; 22.1%) confidence, 99.5% and 93.5% of the videos were scored correctly. From the nCLE sequences that were rated with ‘poor’ confidence (n=20; 7.1%), only 60% of the videos were scored correctly. The overall agreement of the five raters was substantial (IOA κ=0.78, 95% CI 0.70 to 0.86). Individual malignancy criteria were recognised with moderate agreement (table 3). Enlarged cells and dark clumps were present in respectively 54% and 48% of the validated videos, while directional streaming was present in 37% of the videos. The calculated IOR by comparing the raters’ performances between the first and second validation session was excellent (κ=0.82±0.05).

**Table 2:** Scoring performances (%) of the blinded raters (n=5) for the presence of nCLE criteria of malignancy or airway/lung parenchyma based on two validation sessions with 28 nCLE videos from 23 patients (total of 280 ratings)

	Sensitivity	Specificity	Accuracy	PPV	NPV
Validation sessions combined	95.5	93.8	95	97.4	89.3
Validation session 1	92.9	97.5	93.6	98.9	83
Validation session 2	99	90	96.4	96.1	97.3

nCLE, needle-based confocal laser endomicroscopy; NPV, negative predictive value; PPV, positive predictive value.

**Table 3:** Calculated IOA and IOR for the final diagnosis and IOA for the individual nCLE malignancy criteria based on the scoring performances of the five raters during both validations sessions

	κ value
Final diagnosis	
IOA	0.78 (0.70–0.86)
IOR	0.82±0.05
Enlarged pleomorphic cells	0.53 (0.44-0.61)
Dark clumps	0.49 (0.41–0.58)
Directional streaming	0.46 (0.38–0.55)

The data are presented as the IOA κ (95% CI) unless stated otherwise. Landis-Koch interpretation system: poor <0.2, fair 0.21–0.4, moderate 0.41–0.6, substantial 0.61–0.8 and excellent 0.81–1.

IOA, inter-observer agreement; IOR, intra-observer reliability was calculated by comparing the first and second validation session.; nCLE, needle-based confocal laser endomicroscopy.

## Discussion

In this prospective trial, we demonstrated that bronchoscopic nCLE imaging of peripheral lung lesions suspected of lung cancer is feasible and safe in selected patients. Importantly, smart needle CLE imaging enables real-time malignancy detection at the needle tip during bronchoscopy. Blinded raters were able to consistently distinguish nCLE videos of lung cancer from airway/lung parenchyma with high accuracy (95%) after a short training. The results of this study demonstrate the potential of nCLE imaging as a real-time bronchoscopic guidance tool to reduce the current near miss rate of sampling peripheral lung lesions.

To the best of our knowledge, this is the first systematic study analysing nCLE imaging in peripheral lung tumours. We demonstrated that nCLE imaging in a peripheral lung tumour allows real-time visualisation of individual malignant cells at the needle tip, enabling tissue acquisition at the optimal position. In a single case, Su *et al* used a similar nCLE approach, however without intravenous fluorescein administration.^20^ An advantage of intravenous fluorescein is that it illuminates the extracellular matrix and blood vessels but not the cells, creating contrast between cells and the fluorescein-rich background.^16^ The nCLE imaging of the tumour without fluorescein use in this previous report revealed black holes but no clear delineation of tumour cells due to the lack of a contrast agent.^20^ Shulimzon and Lieberman performed transthoracic CT-guided nCLE imaging in five patients with parenchymal lung tumours but were unable to visualise individual malignant cells despite fluorescein use,^21^ possibly due to technical reasons or learning curve issues.

Initial experience with CLE imaging for the diagnosis of lung cancer was gained by advancing the CLE miniprobe trough the working channel of the bronchoscope and scanning the surface of the airway wall and lung tumours (probe-based CLE (pCLE) imaging). Although some indirect abnormal pCLE patterns in patients with lung cancer have been described (dark hollows, elastin disorganisation and cellular infiltrates), pCLE imaging was unable to directly visualise the individual tumour cells despite fluorescein use.^22–26^ We attribute this lack of visualisation to the limited penetration depth of the surface-scanning approach and the thickness of the image plane (50 µm) of the CLE probe (AlveoFlex) that causes overlap of cells. With the development of a smaller CLE probe (AQ-Flex), needle-based CLE-imaging within a tumour—as opposed to surface based pCLE imaging—became possible.

Wijmans *et al* performed for the first time needle-based CLE-imaging in central lung tumours and metastatic lymph nodes during endosonographic ultrasound procedures and identified three nCLE lung cancer criteria (enlarged pleomorphic cells, dark clumps and directional streaming).^16^ In the present study we evaluated these criteria for the first time in a prospective manner and no additional nCLE criteria for peripheral lung cancer were observed. In accordance with Wijmans’ findings, the criterium ‘enlarged pleomorphic cells’ was present in all imaged lung tumours while the criteria ‘dark clumps’ and ‘directional streaming’ were variably present. The feature ‘directional streaming’ was remarkably more often present in the present study as compared with Wijmans’ study (11% vs 37%); an explanation might be that this phenomenon is more often present in lung tumours than in metastatic lymph nodes.

In the present study, we proposed four nCLE criteria for airway and lung parenchyma. We showed that autofluorescent elastin fibre bundles and alveoli can be visualised with nCLE imaging as was reported in previous studies performing pCLE imaging.^23 25 27^ The nCLE criteria ‘still image’ and ‘bronchial epithelium’ have not been described yet. Studies performing pCLE imaging in the airways were unable to visualise the bronchial epithelium due to the limited penetration depth of the pCLE approach.^26–28^ By performing needle-based CLE-imaging we punctured the epithelial layer and in vivo visualised the bronchial epithelium. We believe the criterium ‘still image’ is the result of the CLE miniprobe being advanced in a larger airway, not touching the airway wall and creating an image without cellular or autofluorescent structures. Since we described these criteria for the first time, additional studies are needed to confirm our findings.

In this study in 20 out of 24 suspected patients with lung cancer, the pathological analysis of TBNA and biopsies established a lung cancer diagnosis. What can we learn from the nCLE imaging in the four patients without bronchoscopic tissue proof of malignancy? In two patients nCLE imaging revealed a malignant pattern and both were later diagnosed with adenocarcinoma. Probably the needle had slightly moved during the retraction of CLE miniprobe before the actual aspirate, despite the fluoroscopic guidance indicating the needle was in the right position. This might have resulted in a slightly different sampling area. For future studies, the systematic use of a guide sheath might prevent the needle to change position during the retraction of the probe. In a single patient, nCLE imaging revealed an alveolar pattern, indicating the needle had not reached the lesion. In the fourth patient, nCLE imaging showed an increased density of small cells but no malignant features. The patient underwent lobectomy and was diagnosed with an organising pneumonia. These four cases demonstrate that microscopically visualising the tissue at the tip of the needle with the nCLE technique might provide additional information in case of a non-malignant tissue sampling.

This study has several limitations. We performed nCLE imaging in a limited number of selected patients with relatively large lesions and a high prevalence of lung cancer. Although nCLE imaging provides high-quality microscopic images in a real-time fashion, its use is inevitably dependent on the guidance tool, such as rEBUS, electromagnetic navigation and robotic bronchoscopy, to bring the needle to the target area. Whether feasibility and outcomes of nCLE imaging in larger, multicentre cohorts with smaller lesions without a bronchus sign and different (benign) pathologies will be similar, has to be evaluated. Another limitation is the selection of video material used in the validation set. Like most published CLE studies, we had to make a selection to make the validation sessions feasible in time. Consequently, limited videos were included in the validation set to validate each individual airway/lung parenchyma criterium.

Strengths of this innovative study are the prospective design, the direct comparison between the nCLE videos and the cytopathology of the corresponding puncture, the strong reference standard and the consistent and highly accurate judgement of the blinded and inexperienced raters. Of every evaluable nCLE-imaged lung lesion at least one video was included in the validation set to make the selection of nCLE videos most representative.

In addition to improving the current diagnostic bronchoscopic procedures and pending technological developments, application of nCLE into robotic bronchoscopic navigational technology might result in an optimal real-time peripheral lung cancer detection tool.^11 29 30^ For future studies, it would be interesting to prospectively use the nCLE malignancy and airway/lung parenchyma criteria to assess the number of needle repositionings based on the nCLE guidance, in order to evaluate the added value of nCLE imaging to adequately sample the peripheral lung tumour. Also will it be of key importance to develop nCLE criteria of benign (eg, granulomas, hamartomas) pathologies in order to make a clear distinction with malignant lung lesions.

In conclusion, we have demonstrated that bronchoscopic nCLE imaging in peripheral lung cancer is feasible, safe and allows real-time malignancy detection at the tip of the needle. Blinded raters were able to make a clear distinction between malignant tissue and airway/lung parenchyma, demonstrating the potential of nCLE imaging as a real-time guidance tool to reduce the bronchoscopic near-miss rate of peripheral lung cancer.

## Data Availability

All data relevant to the study are included in the article or uploaded as supplementary information. Needle-based confocal laser endomicroscopy video data and source data of the statistical analysis are available upon reasonable request.
